# The Value of a Nursing Certificate

**Published:** 1920-08-14

**Authors:** 


### The Value of a Nursing Certificate.

*To the Editor of* The Hospital.

Sir,—It is now recognised that hospitals are not attracting the best type of woman for training as a nurse, also that they are not attracting enough women of any suitable type to fill ordinary vacancies, much less to increase the staff sufficiently to grant the eight-hour day that committees generally have consented to give. There is also a growing tendency amongst the better-educated women who trained some years ago to desert the institutions they have loved, not only for work outside hospitals, but for work outside the nursing profession altogether. This state of affairs is usually ascribed by the public, by hospital managers, and by the medical profession, to three definite causes—viz.: inadequate pay, long hours of duty, and the number of other occupations now open to women. These causes, undoubtedly, have been responsible for the shortage to a very great extent, but there are others that should not be overlooked. Amongst them may be named the dislike of the present-day young woman for domestic work of any kind. The work of a sick nurse, being of an essentially domestic nature, is therefore avoided. Another is the very small value that now attaches to a nursing certificate when, after three or four years of work and study, it is at last obtained.

The medical student must qualify and obtain his diploma before being allowed to specialise in any particular branch of his profession, and no public authority will employ him unless he has done so. By this means the standard of the medical profession is kept up. When dealing with the nursing profession the authorities do not insist on a general nursing diploma before special work is undertaken; in fact, the tendency is entirely in the opposite direction. Public authorities are found employing untrained or partially trained women as health visitors, etc. It has now become the exception rather than the rule for the hospital masseuse to be a trained nurse. Untrained or partially trained women are employed in private hospitals and nursing homes. The newly certificated nurse asks ruefully why she should have spent three or four years in general training only to find that in any special branch of her work she stands on the same footing as the untrained woman, and only in hospital (where no large proportion can be retained) is her certificate considered valuable. If she wishes to take up health-visiting she must take the same technical training as the untrained woman. She hears that a diploma for general training is not essential for public health work. She never hears of a medical man or woman being accepted for public-health work unless fully qualified beforehand. The nurse who wishes to take up massage (another special branch of nursing) is told the same story. Her certificate does not count. She is again in the same position as the untrained woman. If she takes up private nursing she may find that her fellow-nurse (receiving the same fees) is an uncertificated woman, with more or less (usually less) experience.

The potential probationer naturally inquires, "Why should I spend three or four years training for a nursing certificate when without doing so I could get a much shorter and less arduous training in some special branch which would ensure to me a better position and better prospects?" It is now more advantageous for a woman of fair education and intelligence to borrow the money to enable her to take a course of special training in health-

**The Editor's Letter Box—(*continued*).**

visiting, massage, or some other special branch of work, than to train to become a skilled nurse. The young woman of the present day keeps her own interests distinctly in view, and although (if her tastes lie in that direction) she will probably take up some work in connection with the care of the sick, she will choose one of those branches that offer the best prospects. The educated woman who trained some years ago and now holds a sister's appointment feels that unless she, too, takes some special training (in which case she will gain, as a rule, no advantage from holding a nurse's certificate), there are only two courses open to her. She may remain a sister and struggle on with the very poor type of probationer who is coming forward, and who cannot be made into the kind of nurse that is likely to be a credit to the profession, or she may become, possibly, a hospital matron, a position to which many no longer aspire owing to the difficulty of obtaining probationers and maids, and the difficulties caused by well-intentioned outside individuals with no practical experience in hospital management.

There are still, as there have aways been, women who have a vocation for nursing and will do it, whatever the conditions or the difficulties, but there are not enough of them to go round—not half enough of them. The others need some inducement to join the ranks of trained nurses. There is none.—Yours faithfully,

